# Reasons for low long-lasting insecticide-treated net use and repurposing: qualitative study from southern Ethiopia

**DOI:** 10.3389/fpubh.2025.1561037

**Published:** 2025-08-21

**Authors:** Misganu Endriyas, Mekidm Kassa, Mintesinot Melka, Agegnehu Gebru, Yilma Chisha

**Affiliations:** ^1^South Ethiopia Public Health Institute, Jinka, Ethiopia; ^2^College of Medicine and Health Sciences, Arbaminch University, Arbaminch, Ethiopia; ^3^JSI Ethiopia, USAID Quality Healthcare Activity, Hawassa, Ethiopia

**Keywords:** LLIN use, low utilization, malaria, reasons, repurposing

## Abstract

**Background:**

Long-lasting insecticidal nets (LLINs) are the main vector control tools and remain protective against malaria, even in the presence of high pyrethroid resistance. However, in sub-Saharan Africa, the estimated percentage of the population sleeping under LLINs is low. Hence, this qualitative study was conducted to explore perceptions about LLINs and the reasons for low LLIN use in southern Ethiopia.

**Methods:**

Qualitative cross-sectional study was conducted in southern Ethiopia. Study areas were selected based on low LLIN use following a quantitative survey. Seven focus group discussions (FGDs) with a total of 52 discussants were conducted. Data were managed manually using Microsoft Word and were analyzed thematically.

**Results:**

The themes that emerged were ownership of LLINs, perceived lifespan of LLINs, uses of LLINs, reasons for LLIN non-use, and recommendations. Participants indicated low LLIN coverage and interrupted maintenance supply. The pattern of LLIN utilization varied between groups, as some said it was improving while others said it was decreasing. The expected life span of LLINs reported varied from a minimum of 3 months to a maximum of 3 years. Discussants from all FGDs described that the possibility of discarding or repurposing LLINs is high when it does not kill mosquitoes. Some discussants added the finding that ineffectiveness was worsened by exposing LLINs to direct sunlight to decrease suffocation. All FGD discussants agreed that the main reason for not using LLINs was a lack of awareness, which in turn caused negligence. Some groups in pastoralist areas added the perception that LLINs do not protect from malaria as a reason for non-use.

**Conclusion:**

The low LLIN use and high repurposing practices were noted due to different reasons, including low awareness, negligence, ineffectiveness of LLINs in killing mosquitoes, and others. LLINs are repurposed mainly for covering different things and making ties. Continuous awareness creation activities and corrective measures might improve LLIN coverage and use.

## Background

Malaria is one of the major global public health problems, with an estimated 263 million cases in 2023. Ethiopia contributed 3.6% of cases and 3.1% of deaths of global malaria in 2023 (1). Although Ethiopia has launched a malaria elimination program, malaria cases have recently been increasing in Ethiopia, and compared to cases in 2022, the number of cases doubled in 2023 (2). The routine health management information system (HMIS) reports also show that the prevalence of malaria is increasing in southern Ethiopia.

Because of the complex nature of malaria epidemiology, the World Health Organization (WHO) recommends combined strategies for malaria prevention and control, of which early diagnosis and prompt effective treatment of malaria and long-lasting insectcide-treated net (LLIN) utilization are the main ones (3–5). The WHO recommends LLINs for every person at risk of contracting malaria, which is adopted in the Ethiopian National Malaria Guideline (6, 7). Between 2000 and 2015, in Africa, LLINs averted an estimated 68% of total cases averted by interventions (663 million) (8). However, in 2021, in sub-Saharan Africa, the estimated percentage of the population with access to LLINs within their household was 54%, and the percentage of the population sleeping under LLINs was only 47% (3).

Factors contributing to low LLIN ownership mainly include allocation efficiency, retention, and durability of the net fabric, while variations in usage are affected by age, season, gender, and malaria risk (3). Education level of household heads, wealth of families, the number of under-five children in the household, knowledge about LLINs, hotness of the weather, abundance of mosquitoes, room designs, color, odor, and shape of LLINs also influence LLIN utilization (9, 10). In Ethiopia, knowledge of malaria and LLINs, perception about LLINs, risk perception about malaria, and perception of low efficacy of LLINs are also important behavioral factors for the utilization of available LLINs (11).

In Ethiopia, community mobilizations are held before the mass distribution of LLINs. In addition, routine health education is part of the health extension program (7). Monitoring and evaluation of the implementation status of the malaria prevention and control program is vital for the national malaria prevention and control program (12). Evidence in Southern Ethiopia suggest that ownership, utilization, and determinants of utilization of LLINs vary significantly over time and place (13). As up-to-date evidence on these indicators is crucial for evidence-based decision-making, the regional health bureau conducted a community survey to assess LLIN ownership, utilization, and its determinants in the Southern Nations, Nationalities, and Peoples’ Region (SNNPR) of Ethiopia (13). Following a quantitative study, detailed qualitative information was required in areas where LLIN utilization was low to better understand why people are not using LLINs. Hence, this qualitative study was conducted to explore perceptions about LLINs and its use in SNNPR.

## Materials and methods

A cross-sectional study using qualitative methods was conducted in 2019. The study was conducted in SNNPR, which was the third largest administrative region of Ethiopia, representing approximately 20% of the country’s population. It was the most diverse region in the country in terms of language, culture, and ethnic background. Currently, the SNNPR is sub-divided into four administrative regions. These are the Sidama, Southwest Ethiopia, South Ethiopia, and Central Ethiopia administrative regions.

The study considered malaria endemicity and LLIN utilization results generated by a quantitative survey (13). Of 1,202 households that owned LLIN(s), only approximately two-thirds (66.0%) of households reported that they slept under LLINs the night preceding the survey. In addition, geographical residence variability (including the urban–rural mix) was also considered. Considering the existing logistics, time, and idea saturation (14), seven focus group discussions (FGDs) were conducted; three in South Ethiopia, two in Central Ethiopia, one in Sidama, and one in Southwest Ethiopia. The participants were members of the community-level Women’s Development Network. Women’s Development Network (sometimes called Women’s Development Team) is the community-level network of women in which a better-performing woman leads an average of 30 neighboring households. It is widely used for health education and demands creation for health services in general, especially maternal and child health services. These women are trained and usually communicate with community health extension workers. Members of the network were considered for the study as they had better information about the community. They acted as key informants and discussed the nature of their network rather than their own experience. Members of these networks were selected in consultation with health extension workers. To minimize bias, health extension workers and women were informed about the objectives of the study and that it was not an evaluation.

The interview guide was prepared in English and translated into Amharic by the investigators. To address the trustworthiness of the interview guide, the developed guide was reviewed by program experts and the research team. A pair of experts involving one moderator and one note taker, who have the master’s degree in public health and experience in qualitative data collection, were trained and conducted the discussions. The sessions took place in the community and were recorded on digital audio recorders. All discussants were given codes before starting the discussion, and these codes were used to facilitate discussion. Sessions, on average, lasted for 1 h. The digital audio records were transcribed verbatim and translated into English.

Framework analysis was used in this study because of its flexibility for inclusion of *a priori* and emerging concepts and application for policy studies (15–17). Deductive and descriptive approaches were used to summarize and describe data (17, 18). Qualitative description is used to describe a situation using data from those experiencing the phenomenon and also when information is sought to improve interventions (19, 20).

The specific steps used to summarize the data were adopted from studies of Gale et.al (17) and Cresswell et.al (21). These steps include (1) preparing the data for analysis, (2) reading all the data (several times to understand and validate the content), (3) coding, (4) generating a description and themes and (5) representing the description and themes (direct quotes that best describe the main themes were reported).

Following these steps, each transcription was read multiple times to become familiar with the content. The analysis was done manually in Microsoft Word. Keywords were color-coded, and similar codes were categorized into groups of similar ideas. Groups of related data were clustered and refined through subsequent revisions to categorize data into similar themes.

The Ethical Review Committee of SNNPR Health Bureau approved the study (ref. no.: 6-19/31111). A letter of permission from the regional health bureau was sent to the study districts. Informed verbal consent was approved and taken from all respondents, and data were handled anonymously.

## Results

Seven FGDs with a total of 52 discussants were conducted: 2 FGDs with 6 discussants each and 5 FGDs with eight discussants each. The themes that emerged to summarize the results were categorized into ownership of LLINs, perceived lifespan of LLINs, uses of LLINs, reasons for LLIN non-use, and recommendations.

### Theme 1: ownership of LLINs

Discussants from each kebele pointed to different times for the last LLIN distribution. Some discussants reported it was 5 years ago, while a few others reported it was being provided for people in need (coverage, maintenance, supply). All discussants complained that they needed supplies as there is a shortage of LLINs, varying from a need for adequacy to non-existence. In addition to the shortage of LLINs, some discussants complained about unfair distributions. Discussants in one FGD complained that the distribution was only for pregnant women, while discussants in another FGD reported that the distribution was based on the number of beds in the house without considering family size.

*“It is too long-ago, 5 years back when bed net was distributed. In addition, it was offered for pregnant women only which was in 2007 E.C (2014 GC) for last time.” [Rural, 35]*

*“If I am not mistaken, LLINs was distributed in 2009 E.C (2016 GC). But it was given by considering the number of beds, not according to the family size.” [Rural, 25]*

*“Distribution is very low though it was done this year. In my village, only one for one lactating mother was distributed this month. If there is any chance to increase the distributions, it would be good to keep the health of community.” [Urban, 45]*

### Theme 2: perceived lifespan of LLINs

The expected life span of LLINs was also reported to vary, ranging from a minimum of 3 months to a maximum of 3 years. The majority of discussants reported a lifetime of 6 months to 1 year and added that it depends on the housing structure and exposure of LLINs to smoke and dust. Discussants from all FGDs described that the possibility of discarding LLINs is high when it does not kill mosquitoes. An FGD discussant also reported that sometimes people put LLINs in direct sunlight to minimize the toxicity of the chemical before using it, and when they take it back, it does not kill mosquitoes, and then, they use it for other purposes. Some say that people discard it when the chemical is weak and thus does not kill mosquitoes, as lice starts to live on LLINs. FGD discussants in pastoralist areas enforced the low lifespan of LLINs by justifying the use of LLINs on unstructured beds and handling it under direct sunlight.

*“We say it can be used up to 3 years. But people, especially in rural areas, discard it because of its reduced effectiveness due to chemical lose. And they use it for other purposes due to low awareness level. They are not willing to use it for more than 3 months.” [Urban, 40]*

*“People in rural areas do not use even for a year. When it becomes dirty with smoke, they do not wash it and use it for other purposes. They discard it as lice enter to bed net. However, some use it properly.” [Urban, 40]*

*“When you use LLINs outside of structured and standardized houses, it is exposed to rain and excessive sunlight and easily tears out. Hence, households perceive that it will no longer use and kill mosquito.” [Pastoralist, 35]*

### Theme 3: uses of LLINs

The uses of LLINs presented here include proper uses and abuses of LLINs other than malaria prevention. All FGDs reported that LLINs prevent malaria by preventing mosquito bites. In addition, all FGDs described the high priority population in the cases of LLINs shortage as pregnant women, under-five children, and lactating mothers, though the majority, except the urban group, did not clearly reveal the reason for prioritization. The pattern of LLIN utilization varied between groups, as some said it was improving while others said it was decreasing.

All FGD discussants reported alternative uses of LLINs besides malaria prevention. These include protection from flies, providing heat, protection from dust, mangling “Kocho” (food prepared from false banana or “enset”), covering latrines; making curtains or screens; making kerchiefs, scarfs, or skirts; making ties or ropes; collecting and tying cereals, fruits, and bran; cleaning house; covering tombs; and protecting chicken and/or hens. Pastoralist participants added that it can also protect scorpions and snakes.

*“We do not have structured houses, and we sleep outside. Hence, we are exposed to scorpions and snakes. If we use LLIN properly, it will protect us from bite by reptiles and venomous insects.” [Pastoralist, 35]*

*“We use LLIN for many other purposes like to cover latrine, to carry water by pot or jerrican, as a rope to tie objects, to cover hen house, to carry fruits from place to place and to cover of tomb.” [Pastoralist, 35]*

*“Sometimes people use LLIN to cover food and some make rope to tie load on donkey. However, after we provide awareness, it (LLIN use) is improving.” [Urban, 55]*

### Theme 4: reasons for non-use

All FGD discussants agreed that the main reason for not using LLINs was a lack of awareness, which in turn caused negligence. Some groups in pastoralist areas added the perception that LLINs do not protect from malaria as their reason for non-use. In both rural and urban settings, some groups listed the smell of chemicals, burning sensation, shape of LLINs, chemical fade-up to kill mosquitoes, low malaria incidence, and poor follow-up of LLINs utilization as reasons for non-use.

*“Some people say, ‘when we see no died mosquito on the bed net, we know that LLINs do not kill mosquito and do not protect malaria’.” [Pastoralist, 35]*

*“When we sleep under LLINs, we feel hot, and it is not convenient to sleep under it.” [Pastoralist, 40]*

*“Due to chemical spray conducted years ago, malaria is getting low, and this makes people to give less attention to LLINs use.” [Urban, 40]*

*“Some people do not use LLINs continuously complaining smell of chemical and fear that it can affect their breathing.” [Urban, 35]*

*“Sometimes the shape of LLINs may hinder its use. People living in rental houses may not be allowed to pierce the wall to fix LLINs. If it [LLINs] is umbrella shaped type [conical], it would be better.” [Urban, 35]*

*“Some of our community members fear that the bed net is treated with toxic substance that would harm them and children. So, they use it after washing.” [Urban, 35]*

### Theme 5: ways forwarded

All FGD discussants indicated that health education is important for improving community awareness. In pastoralist FGDs, participants described that the number of health extension workers (community-level healthcare workers assigned at health post) is not adequate to reach the community and added that Women’s Development Network leaders should be trained to train the community. Some groups also supplemented periodic follow-up of LLIN utilization with corrective measures (punishment) on households that do not use LLINs properly and reward people for using them properly. Regarding LLIN coverage, all FGD discussants requested resupply, some preferring the conical shape because of its ease of fixing. Some added periodic treatment of LLINs with chemicals.

## Discussion

The themes used to summarize the results were categorized into the ownership of LLINs, the perceived lifespan of LLINs, uses of LLINs, reasons for LLIN non-use, and recommendations.

The WHO recommends the use of LLINs by every person in malaria-endemic countries at risk of contracting malaria (3, 22), as LLINs remain protective against malaria, even in the presence of high pyrethroid resistance (3) by blocking exposure to potentially infective mosquito bites (4, 5). However, cohort studies in Ethiopia indicated that LLINs last shorter than the expected 3 years due to high attrition and loss of integrity, resulting in low coverage and utilization (23, 24).

Factors affecting universal LLIN ownership and use include allocation efficiency, retention, and durability of the net fabric. The retention of LLINs is determined by the household’s attitudes and behaviors toward their nets (3). Systematic reviews conducted on the use of LLINs in sub-Saharan Africa have reported that the education level of household heads, family wealth, the number of under-five children in the household, knowledge that sleeping under a mosquito net protects against malaria, cost of LLINs, hot weather, abundance of mosquitoes, room designs, color, odor, and shape of LLINs influence LLIN utilization (9, 10).

In this study, all FGD discussants agreed that the main reason for not using LLINs was a lack of awareness, which, in turn, leads to negligence. The other reasons for not using LLINs include the use of chemicals that do not kill mosquitoes in LLINs, the perception that LLINs do not protect from malaria, the smell of chemicals, burning sensation, the shape of LLINs, low malaria incidence, and poor follow-up from the government side. A study conducted in northwest Ethiopia also noted that the perception of the ineffectiveness of LLINs in killing mosquitoes was a major barrier to the persistent use of LLINs (25). In line to this study, it was noted in discussion that people put LLINs in direct sunlight to minimize suffocation from new LLINs, which degrades the pyrethroid insecticides used on LLINs (26).

Misuses of LLINs are common in Ethiopia and is often associated with local needs (25). All FGD discussants reported misuses of LLINs or unintended uses of LLINs other than malaria prevention and control, mostly for covering and tying purposes such as covering latrines, tombs and chickens, mangling “Kocho,” and making ropes. Because of these reasons and repurposing, a significant number of study participants reported that LLINs lasted less than 3 years, including a few (3–6) months. Not using or repurposing the LLINs that do not kill mosquitoes is a crisis for the malaria program, both financially and in meeting disease prevention and control goals. Some FGD participants, thus, suggested close follow-up from the government side and corrective measures against LLIN abuses.

The quantitative survey conducted prior to this qualitative study (13) revealed that approximately one third (29.1%) of the participants were not using LLINs, justifying themselves by stating that there was no malaria. Households in moderate malaria burden areas and areas included in the national malaria elimination program (low malaria burden areas) were less likely to utilize LLINs as compared to households in high burden areas. It was also reported that the burden of malaria forces people to use LLINs or not. This might be due to the fact that low perceived risk of malaria reduces LLIN use (27). In Ethiopia, since the alternative vector control, Indoor Residual Spraying (IRS), is not recommended in low malaria stratum settings (28, 29), this should be taken into account to improve consistent LLIN utilization for the success of the program.

This qualitative study mainly focused on areas with low utilization of LLINs and was limited in addressing areas with better LLIN use. Thus, the results indicate critical gaps in LLIN use and present more misuse. In addition, since the area is diverse in socio-cultural and development aspects, the findings may not fully represent the dynamics of LLIN use and repurposing. Moreover, the study participants were women, and the study was limited in addressing perceptions of men. Furthermore, detailed sociodemographic characteristics of the participants were not documented.

## Conclusion

The low LLIN use and high repurposing practice were noted due to different reasons, including low awareness, negligence, ineffectiveness of LLINs in killing mosquitoes, and others. LLINs are repurposed mainly for covering different things and making ties. Continuous awareness creation activities and corrective measures might improve LLIN use.

## Data Availability

The original contributions presented in the study are included in the article/Supplementary material, further inquiries can be directed to the corresponding author.
