# Supplementary material for: Reasons for low long-lasting insecticide-treated net use and repurposing: qualitative study from southern Ethiopia
Source: Front Public Health. 2025 Aug 21;13:1561037. doi: 10.3389/fpubh.2025.1561037 (PMC12408592; doi:10.3389/fpubh.2025.1561037)
Supplement: Supplementary file 1 [file Data_Sheet_1.pdf]

Ownership and utilization of long-lasting insecticide treated nets and associated factors in SNNPR

Guides for discussion

1. How you evaluate ownership ITN in your locality?
   a. How much of households in your locality own ITN?
   b. When was the last supply of ITN?
   c. How was the distribution of last supply of ITN, was that 100%?
2. What do you think about the life span of ITN?
   a. What is the expected life span of ITNs in years?
   b. Why the life span of ITNs is too short?
3. What are the uses of ITN you think of?
4. In addition to the discussed uses of ITN, for what other purposes (misuses) ITN can be used?
5. How do you evaluate the utilization of ITN among households in your locality?
   a. Which population group uses it more and why?
6. Why do you think that some households don't utilize ITN?
7. What should be done to improve ITN utilization in your locality?
